# Persistence of antibiotic resistance genes in beef cattle backgrounding environment over two years after cessation of operation

**DOI:** 10.1371/journal.pone.0212510

**Published:** 2019-02-15

**Authors:** Getahun E. Agga, Kimberly L. Cook, Annesly M. P. Netthisinghe, Rebecca A. Gilfillen, Paul B. Woosley, Karamat R. Sistani

**Affiliations:** 1 Food Animal Environmental Systems Research Unit, Agricultural Research Service, United States Department of Agriculture, Bowling Green, Kentucky, United States of America; 2 Department of Agriculture, Western Kentucky University, Bowling Green, Kentucky, United States of America; Westen University of Health Sciences, UNITED STATES

## Abstract

Confined animal feeding operations can facilitate the spread of genes associated with antibiotic resistance. It is not known how cattle removal from beef cattle backgrounding operation affects the persistence of antibiotic resistance genes (ARGs) in the environment. We investigated the effect of cessation of beef cattle backgrounding operation on the persistence and distribution of ARGs in the beef cattle backgrounding environment. The study was conducted at a pasture-feedlot type beef cattle backgrounding operation which consisted of feeding and grazing areas that were separated by a fence with an access gate. Backgrounding occurred for seven years before cattle were removed from the facility. Soil samples (n = 78) from 26 georeferenced locations were collected at the baseline before cattle were removed, and then one year and two years after cattle were removed. Metagenomic DNA was extracted from the soil samples and total bacterial population (16S rRNA), total *Enterococcus* species and class 1 integrons (*int*I1), and erythromycin (*erm*B and *erm*F), sulfonamide (*sul*1 and *sul*2) and tetracycline (*tet*O, *tet*W and *tet*Q) resistance genes were quantified. Concentrations of total bacteria, *Enterococcus* spp., class 1 integrons, and ARGs were higher in the feeding area and its immediate vicinity (around the fence and the gate) followed by a gradient decline along the grazing area. Although the concentrations of total bacteria, *Enterococcus* spp., class 1 integrons and ARGs in the feeding area significantly decreased two years after cattle removal, their concentrations were still higher than that observed in the grazing area. Higher concentrations over two years in the feeding area when compared to the grazing area suggest a lasting effect of confined beef cattle production system on the persistence of bacteria and ARGs in the soil.

## Introduction

The use of antibiotics in food animal production for the purposes of disease prevention, control or treatment undoubtedly plays a significant role for animal health and welfare, food safety and security, and the public health. With increased demand for animal products, antibiotic use in food animals is projected to increase by 67% in the next two decades [1]. In the United States (U.S.) alone approximately 14 million kilograms of antibiotics were sold in 2016 for intended use in food-producing animals, of which the medically important antimicrobials, as defined by the U.S. Food and Drug Administration (FDA) [2, 3], accounted for 60% of all antimicrobials approved for use in food-producing animals [4]. The FDA estimate also shows that in 2016, 43% (3.6 million kilograms) of medically important antimicrobials and 55% (3.1 million kilograms) of non-medically important antimicrobials of the domestic sales and distribution were intended for use in cattle (combining both beef and dairy cattle). These estimates include all uses (growth promotion, disease prevention or control and therapeutic), and the impact of the recently implemented action to withdraw the use of medically important antibiotics for growth promotion, and to limit all other uses of medically important antibiotics under veterinarian supervision as stated in the Guidance for Industry (GFI #209) by FDA [5] in accordance with GFI #213 [3] is to be seen.

It is estimated that about 20 to 80% of antibiotics administered to animals are excreted either as the parent compound or as metabolites in the urine and feces [6]. Excreted antibiotics can persist in the environment and select for resistant bacteria in the soil even at a lower concentration. Horizontal transfer of ARGs in the environment further disseminates antibiotic resistance [7, 8]. There is unquantified risk that antibiotic traces in animal manure will enrich antibiotic resistant bacterial pathogens and potentially cause subsequent health issues both in humans and animals. Humans can be exposed to resistant bacteria of animal origin through contaminated food products, direct contact with infected animals, or through environmental exposure [6]. However, there is insufficient data to quantify the total public health burden of antibiotic use in food animal production [9]. The impact of antibiotic resistance due to the use of antibiotics in food animal production on public and animal health has been studied more widely. However, it is very recently that the environmental dimension of antimicrobial resistance has been recognized in the “One Health” framework as necessary to address the impact of agricultural use of antibiotics on environmental health [10]. Antibiotic resistance genes have been recognized as environmental contaminants [11, 12].

Beef cattle backgrounding is an intermediate stage between cow-calf and feedlot (finishing) operations in the U.S. beef cattle production system in which weaned calves are managed for a period of time until they attain a desired body weight and body condition before being transferred to feedlots for finishing [13, 14]. Backgrounding typically involves maximal use of pasture and forages with protein and mineral supplements [15]. Confined animal feeding operations can facilitate the transmission and spread of antibiotic resistant bacteria and associated antibiotic resistance genes (ARGs) [16]. However, there is scarcity of information on how livestock production systems affect the level and persistence of antimicrobial resistance in the environment. Specifically, the persistence of ARGs in beef cattle backgrounding operations after animals are removed is not widely studied. It is not known how removal of cattle from a backgrounding operation would affect the persistence of ARGs in the environment previously impacted by animals. The objective of this study was to investigate the effect of destocking on the persistence and distribution of ARGs and bacteria in beef cattle pasture-feedlot type backgrounding environment. We quantified the concentrations of total bacteria (16S rRNA), total *Enterococcus spp*. (23S rRNA), integrase gene (*int*I1) of class 1 integron, erythromycin- (*erm*B and *erm*F), sulfonamide- (*sul*1 and *sul*2), and tetracycline-resistance genes (*tet*O, *tet*Q and *tet*W).

## Materials and methods

### Experimental setup and sample collection

The study was conducted at Western Kentucky University Agricultural Research and Education Complex Beef Cattle Backgrounding Unit in Bowling Green Kentucky. The facility was a semi intensive beef cattle backgrounding operation that consisted of a covered barn with an outdoor feeding and watering (designated as FD), and grazing (designated as GR) areas. The FD and GR areas were physically separated by a fence with an access gate through which calves could move freely between the feeding and the grazing areas for feeding, grazing, watering and resting. Backgrounding occurred for seven years before cattle were removed from the facility in 2010. After cessation of the backgrounding operation, manure was removed by scraping in March 2010 and the site was used for hay production. Hay was harvested during May to November 2011. Detailed description of the backgrounding facility and management practices were previously reported [17, 18]. Preventive health management practices such as vaccinations (against respiratory infections, tetanus and clostridial infections), deworming, castration, ear tag for identification, and implantation with growth hormones were conducted according to the husbandry practices of the backgrounding operation. Sick calves were individually treated with injectable antibiotics including ceftiofur, tilmicosin, enrofloxacin and florfenicol.

**Fig 1:**
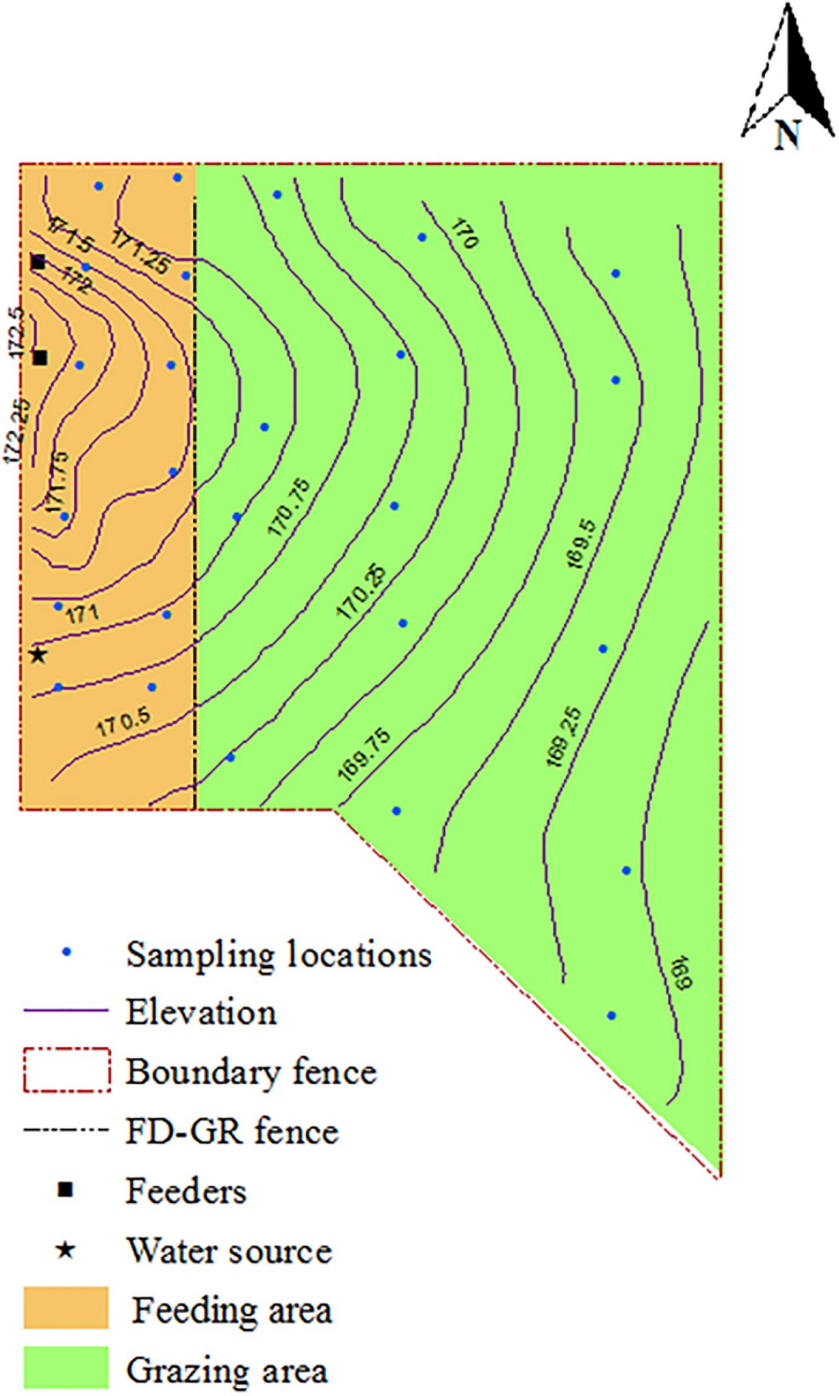
Georeferenced soil sampling locations at beef cattle backgrounding environment monitored for the persistence of antibiotic resistance genes for two years after cessation of operation. The facility was divided into feeding (FD) and grazing (GR) areas separated by a fence.

Soil samples were collected from georeferenced locations (12 locations in the feeding area and 14 locations in the grazing area) as shown in Fig 1. Soil samples were collected from 0–15 cm depth cores by using soil probes sanitized with 70% ethanol between sampling locations. The same georeferenced locations were used for soil sampling in 2010, 2011 and 2012. Throughout the study, soil samples were collected in March of each year to avoid any effect of seasonal variations on the results. The 2010 samples were collected while animals were on the facility and served as baseline samples. The 2011 and 2012 samples were collected after cattle were removed from the facility to evaluate spatial and temporal changes in the concentration of antibiotic resistance genes one- and two-years after cattle removal from the backgrounding operation. The three annual samples also represent an intervention strategy to reduce the level of ARGs that persist in the environment. The 2010 soil samples were collected before manure removal and hay harvesting; the 2011 samples were collected one year after manure was removed; and the 2012 samples were collected following hay harvesting.

### Metagenomic DNA extraction and quantitative PCR

Metagenomic DNA was extracted from 300 mg of soil using the FastDNA Spin kit for soils (MP Biomedical, Santa Ana, CA) according to the manufacturer’s instructions. Real time quantitative PCR (qPCR) was run on Bio-Rad CFX 96 real-time PCR detection system (Bio-Rad, Hercules, CA) to quantify the concentrations of the targeted genes by using published primers, probes and protocols (Table 1). The primers were obtained from Sigma-Genosys (The Woodlands, Texas), and the dual-labeled black hole quencher probes were prepared by Biosearch Technologies, Inc. (Petaluma, CA). The qPCR assay was performed in Qiagen HotStarTaq master mix (Qiagen, Valencia, CA) in a total reaction volume of 25 μL. The assay consisted of 3 mM MgCl_2_, 600 nm each of the forward and reverse primers, 200 nm of probe, and 10 ng of sample DNA or the standard (ranging from 10^2^ to 10^8^ copies). Sample DNA was diluted in 1:500 ratio to reduce the effect of potential PCR inhibitors in the soil. A total of 5 μL of the diluted sample DNA and 5 μL of standard DNA were used as a template in the qPCR reaction.

**Table 1:** Sequences, target size and melting temperature of primers used.

Organism or group	Target gene	Primer	Primer sequence (5’-3’)^†^	Tm. (°C)^‡^	Product size (bp)^§^	Assay type^¶^	Reference
**All bacteria**	16S rRNA	1055-F	ATG GCT GTC GTC AGC T	58	337	TaqMan	[32]
		1392-R	ACG GGC GGT GTG TAC				
		B16s-Taq115-F	FAM-CAA CGA GCG CAA CCC-BHQ				
***Enterococcus* species**	23S rRNA	ECF-748F	AGA AAT TCC AAA CGA ACT TG	60	106	TaqMan	[33]
		ENR-854R	CAG TGC TCT ACC TCC ATC ATT				
		Enterococci-Gl813tQ	FAM-TGG TTC TCT CCG AAA TAG CTT TAG GGC TA-BHQ				
**Class 1 integrons**	intI1	intI1-F	CCT CCC GCA CGA TGA TC	60	280	SYBR	[34]
		intI1-R	TCC ACG CAT CGT CAG GC				
**Erythromycin resistance**	ermB	ermB-91F	GAT ACC GTT TAC GAA ATT GG	58	364	TaqMan	[35]
		ermB-454R	GAA TCG AGA CTT GAG TGT GC				
		ermB-P	FAM-GGG CAT TTA ACG ACG AAA CTG GCT-BHQ				
	ermF	ermF-189F	CGA CAC AGC TTT GGT TGAAC	58	309	SYBR	[35]
		ermF-497R	GGA CCT ACC TCA TAG ACA AG				
**Sulfonamide resistance**	sul1	sul1-F	CGC ACC GGA AAC ATC GCT GCA C	56	163	SYBR	[36]
		sul1-R	TGA AGT TCC GCC GCA AGG CTC G				
	sul2	sul2-F	CGC ACC GGA AAC ATC GCT GCA C	61	190	SYBR	[36]
		sul2-R	TGA AGT TCC GCC GCA AGG CTC G				
**Tetracycline resistance**	tetO	tetO-F	ACG GAR AGT TTA TTG TAT ACC	58	170	SYBR	[37]
		tetO-R	TGG CGT ATC TAT AAT GTT GAC				
	tetQ	tetQ-F	AGA ATC TGC TGT TTG CCA GTG	59	166	SYBR	[37]
		tetQ-R	CGG AGT GTC AAT GAT ATT GCA				
	tetW	tetW-F	GAG AGC CTG CTA TAT GCC AGC	59	168	SYBR	[37]
		tetW-R	GGG CGT ATC CAC AAT GTT AAC				

^†^Probe sequences contained a 5' FAM fluorophore and 3' black hole quencher combination, probe concentration of 100nM, primer concentration of 600nM;

^‡^Tm. (°C) is the annealing temperature of the PCR reaction;

^§^Product size refers to the expected amplification product size in nucleotide base-pairs (bp);

^¶^Refers to type of PCR assay used: TaqMan and SYBR are quantitative real-time PCR assays

16S rRNA and *Enterococcus* spp. were chosen as indicators for total bacteria and gram-positive bacterial populations respectively based on previous reports involving environmental samples in which *E*. *coli* were detected at low concentrations [17, 19]. Class 1 integron integrase gene is considered a potential marker of anthropogenic pollution in the environment due to its widespread distribution in diverse bacterial populations. The genetic linkage of the *int*I1 to ARGs suggests that the integron may serve as a marker for horizontal transfer for ARGs. Therefore, the concentration of *int*I1 should reflect the response of the bacterial community to general selection pressure imposed by anthropogenic pollution [20]. The targeted ARGs confer resistance to macrolides (*erm*B and *erm*F), sulfonamides (*sul*1 and *sul*2), and tetracyclines (*tet*O, *tet*W and *tet*Q) which are the three most commonly used antimicrobial classes in U.S. beef production system [21]. Macrolides are classified as critically important classes of antimicrobials for human medicine, and sulfonamides and tetracyclines are regarded as highly important classes of antimicrobials according to World Health Organization’s ranking of medically important antimicrobials [22]. The *erm* (erythromycin rRNA methylase) genes *erm*B and *erm*F confer resistance to macrolides, lincosamides and streptogramins (MLS), are commonly carried in a wide range of bacterial genera [23] and have been reported from beef cattle and from environments associated with beef cattle production [24, 25]. The *int*I1, *sul*1 and *sul*2, *erm*B and *erm*F are suggested for monitoring human impacts on the environments [26, 27]. The *tet*O, *tet*W and *tet*Q are ribosomal protection genes and have been reported from environments affected by livestock production including soil [28, 29]. As opposed to *tet*A and *tet*B genes which are predominantly detected in Gram-negative bacteria, the *tet* genes (*tet*O, *tet*W and *tet*Q) we targeted in this study have been commonly reported in both Gram-negative and Gram-positive bacteria [30]. Therefore, the three *tet* genes we targeted could capture a more mixed bacterial community in the metagenomic DNA obtained from the soil. The *tet*(O) gene has been found in 35 genera (18 Gram-positive and 17 Gram-negative genera), while the *tet*(W) gene has been identified in 32 different genera (10 Gram-positive and 22 Gram-negative genera). The *tet*(Q) gene has been found in 19 genera (8 Gram-positive and 11 Gram-negative genera). Previous study showed that *tet*A and *tet*B together account for over 99% of tetracycline resistance in *E*. *coli* [31]. Roberts and Schwarz, 2016 [30] also suggest that *tet*(B) gene should be included in any assay if there is interest in determining the Gram-negative tetracycline gene efflux levels in an environmental sample.

### Soil nutrient analysis

Soil samples were air dried and ground to pass through a 2-mm sieve for the quantification of total carbon (C), total nitrogen (N), ammonium nitrogen (NH_4_-N), and nitrate nitrogen (NO_3_-N) as previously described [17, 18]. Briefly, NH_4_-N and NO_3_-N contents were measured by potassium chloride extraction and flow-injection colorimetric analysis with cadmium reduction method on a Lachat Quickchem FIA+8000 analyzer (Hach Company; Loveland, CO) according to the manufacturer’s instructions. Total C and N contents in the soil samples were measured by high temperature combustion in a Vario MAX C-N analyzer (Elementar Americas Inc.; Mt. Laurel, NJ) using a 2-g soil sample.

### Statistical analysis

The gene copy numbers were derived from a 5 μL of the soil microbial community DNA used in the qPCR reaction. The gene quantities per μL were back calculated to obtain gene copies per gram of wet soil. Raw quantities were transformed to log_10_ gene copies/g of soil before analysis. Spatial distribution maps were generated by ArcGIS 10.1 (Environmental Systems Research Institute; Redlands, CA) to visualize the effects of feeding and grazing areas over three sampling time points on the concentrations of total bacteria, total *Enterococcus* species, class 1 integrons and the ARGs across the backgrounding environment. Generalized estimating equations (GEE) with identity link and Gaussian distribution was used to generate a population averaged model with an autoregressive correlation structure of order 1 to compare the gene copies between feeding and grazing areas over three annual sampling time points. The model accounts for the repeated measurements of the spatially defined sampling locations measured at three sampling time points. Model adjusted expected marginal mean gene copies were plotted against sampling year. A contrast for differences in the mean gene copies was used to examine for any significant change in the mean gene copies one- and two-years after cattle were removed relative to the 2010 baseline level. The effect of soil nutrients on the concentrations of the measured outcomes were analyzed by linear regression model in a GEE framework. All statistical analyses were conducted in STATA 15.1 (StataCorp, College Station, Texas).

## Results

### Spatial and temporal variations in the concentrations of total bacteria, total enterococcus species and class 1 integrons

**Fig 2:**
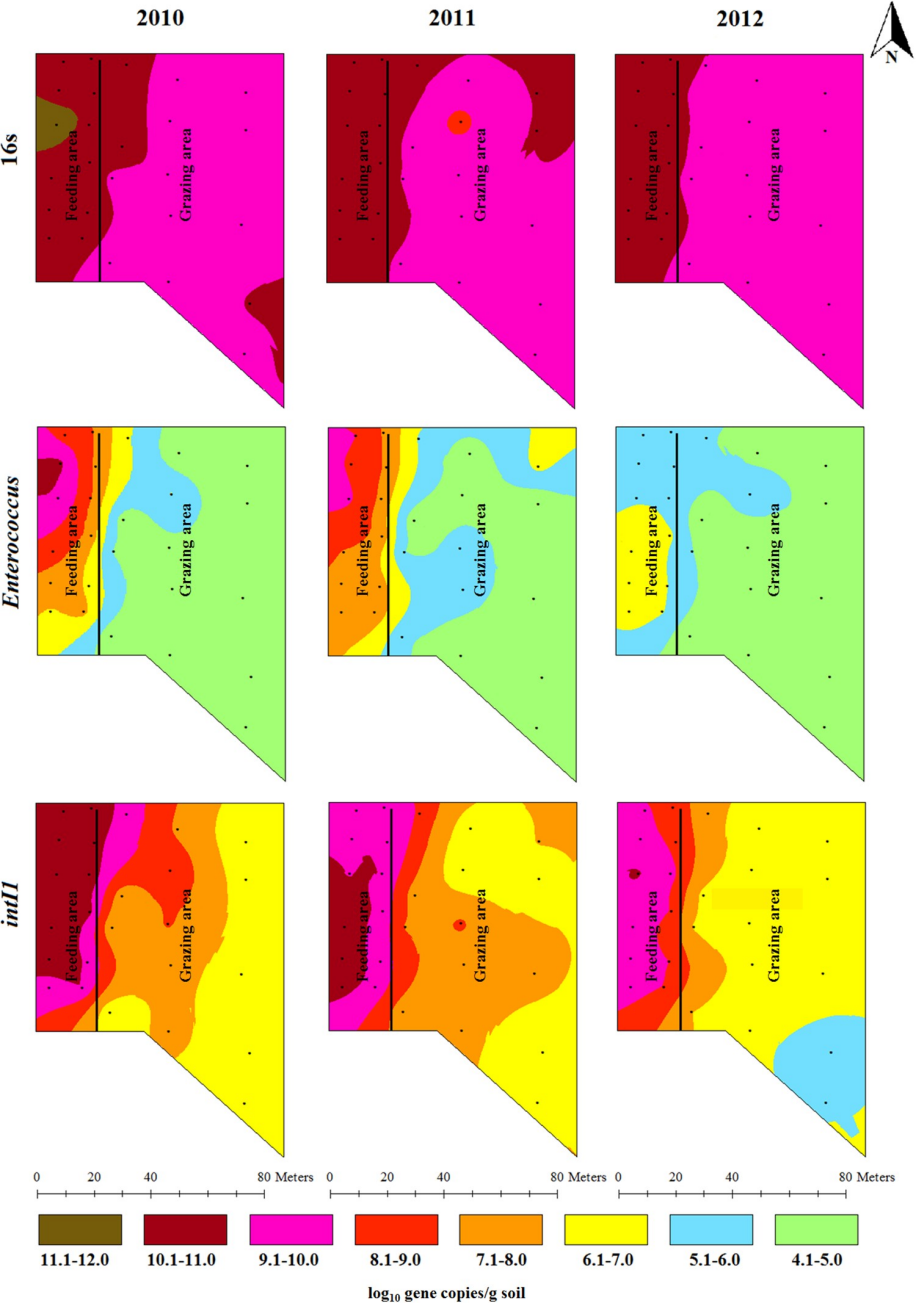
Spatial distributions of total bacteria, total *Enterococcus* species and integrase gene of class 1 integrons in beef cattle backgrounding environment over two years period after cattle removal.

The total bacterial spatial distribution pattern shows higher bacterial concentrations (>10 log_10_ copies/g soil) in the feeding area and around the fence, when compared to the grazing area during the three sampling times. In 2011, high total bacterial concentrations were observed around the fence, gate area and along the gate flow in the grazing area. This high concentration area was diminished in 2012 compared to the 2011 (Fig 2). Statistical and temporal analyses showed that the mean concentrations of total bacteria at the three yearly sampling points were significantly (*P* < 0.05) higher in the feeding area compared to the grazing area (S1 Fig). The mean concentration of total bacteria did not significantly (*P* > 0.05) change one year after cattle were removed compared to the 2010 baseline level. However, two years after cattle were removed the mean concentration of total bacteria significantly (*P* < 0.05) reduced (0.2 log_10_ gene copies/g of soil reduction) particularly in the feeding area (S1 Fig; Table 2).

**Table 2:** Change in log_10_ copies of antibiotic resistance genes over two years after cessation of the beef cattle backgrounding operation compared to 2010 level.

Gene target	Year	Feeding area				Grazing area			
		Contrast	95% confidence interval		*P*-value	Contrast	95% confidence interval		*P*-value
**16S rRNA**	2011	0.02	-0.17	0.21	0.835	-0.08	-0.27	0.10	0.391
	2012	-0.21	-0.40	-0.03	**0.025**	-0.21	-0.39	-0.02	**0.028**
**Enterococcus species**	2011	0.11	-0.39	0.61	0.679	0.30	-0.19	0.79	0.233
	2012	-2.06	-2.55	-1.57	**<0.001**	0.02	-0.47	0.51	0.936
***int*I1**	2011	-0.23	-0.69	0.23	0.334	-0.16	-0.62	0.29	0.47
	2012	-1.13	-1.58	-0.68	**<0.001**	-0.76	-1.21	-0.31	**0.001**
***erm*B**	2011	0.32	-0.23	0.87	0.251	0.57	0.04	1.11	**0.035**
	2012	-1.01	-1.55	-0.48	**<0.001**	0.79	0.25	1.32	**0.004**
***erm*F**	2011	-0.09	-0.61	0.42	0.721	-0.25	-0.77	0.26	0.339
	2012	-1.46	-1.97	-0.96	**<0.001**	-0.59	-1.12	-0.07	**0.028**
***sul*1**	2011	-0.18	-0.59	0.22	0.374	-0.11	-0.50	0.29	0.593
	2012	-1.00	-1.40	-0.61	**<0.001**	-0.63	-1.02	-0.23	**0.002**
***sul*2**	2011	-0.04	-0.52	0.43	0.851	-0.39	-0.85	0.07	0.097
	2012	-1.01	-1.5	-0.55	**<0.001**	-0.25	-0.71	0.21	0.288
***tet*O**	2011	-0.13	-0.48	0.22	0.473	0.46	0.12	0.80	0.009
	2012	-1.16	-1.51	-0.82	**<0.001**	-0.18	-0.53	0.16	0.301
***tet*Q**	2011	0.11	-0.48	0.70	0.720	0.28	-0.85	0.30	0.348
	2012	-1.31	-1.88	-0.73	**<0.001**	-0.67	-1.25	-0.09	**0.023**
***tet*W**	2011	0.20	-0.26	0.67	0.387	-0.01	-0.46	0.45	0.976
	2012	-1.00	-1.45	-0.55	**<0.001**	-0.23	-0.68	0.23	0.324

P-values for statistically significant differences in the mean concentrations of the genes measured in 2011 and 2012 compared to 2010 are shown in bold.

The concentration of total *Enterococcus* spp. showed many concentration gradients indicative of concentration variability across the backgrounding landscape with seven concentration ranges observed in 2010, six in 2011 and three in 2012 showing a decline in the total *Enterococcus* spp. population in space and time. High concentrations of total *Enterococcus* spp. were observed around a feeder in the feeding area in 2010. The concentration gradually diminished in locations farther from the feeders and waterers as well as over time (Fig 2). Mean concentration of total *Enterococcus* spp. did not significantly (*P* > 0.05) change one year after cattle were removed compared to the 2010 baseline level (S1 Fig; Table 2). Two years after cattle were removed from the backgrounding facility, a statistically significant (*P* < 0.05) reduction was observed in the mean concentration of total *Enterococcus* spp. (2 log_10_ gene copies/g of soil reduction) compared to their 2010 levels in the feeding area (Table 2). The *int*I1 concentration showed similar trend as the total bacterial population except that it showed more variable concentration ranges over the backgrounding landscape as compared to mostly two concentration ranges seen for the total bacterial concentration. The *int*I1 concentration declined in time and space in a gradient manner (Fig 2). The mean concentration of *int*I1 did not significantly (*P* > 0.05) change one year after cattle were removed compared to the 2010 baseline level (S1 Fig; Table 2). Two years after cattle were removed from the backgrounding facility, a statistically significant (*P* < 0.05) reduction was observed in the mean concentration of *int*I1 (1 log_10_ gene copies/g of soil reduction) compared to their 2010 levels in the feeding area (Table 2).

### Spatial and temporal variations in the concentrations of antibiotic resistance genes

**Fig 3:**
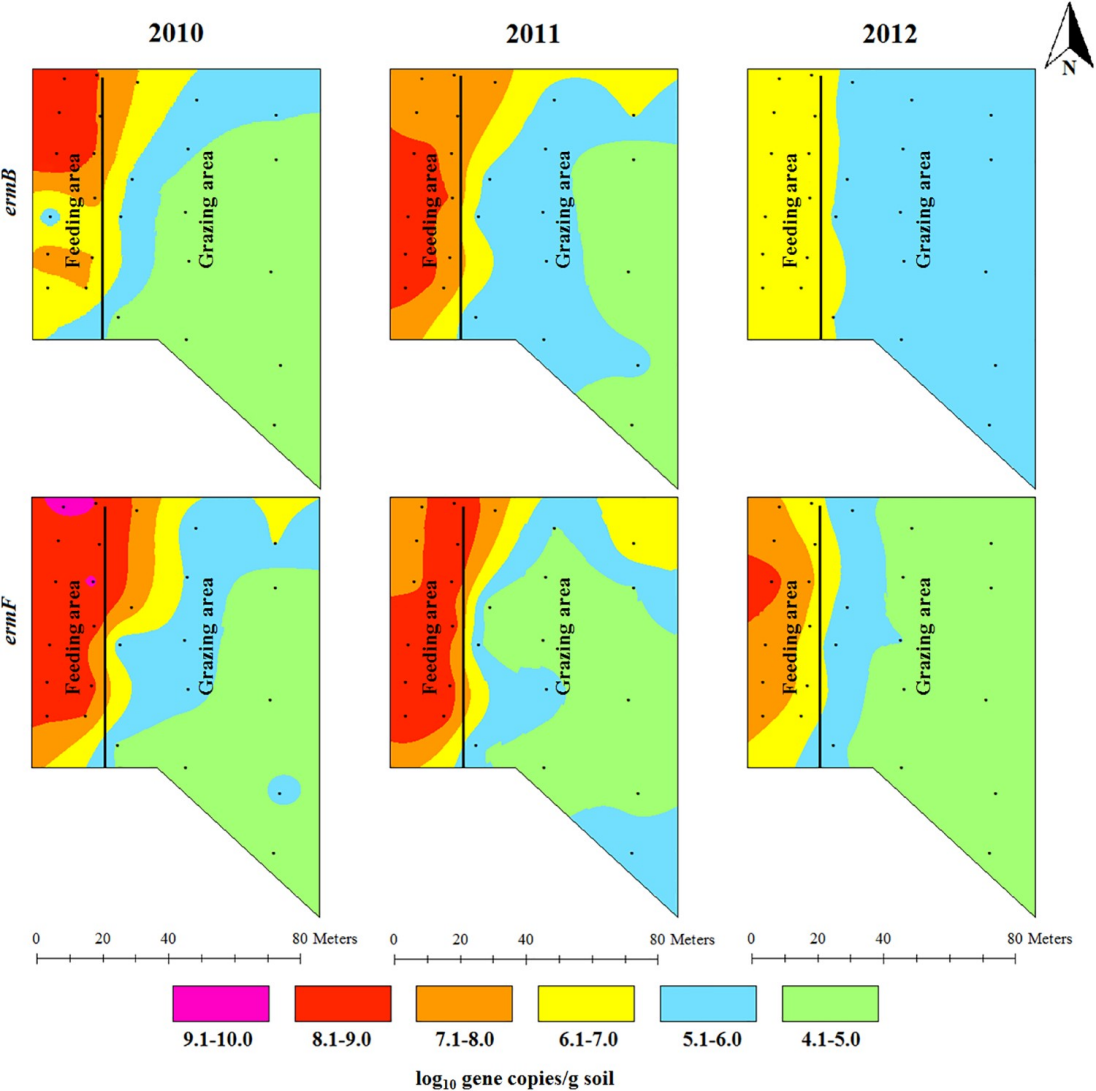
Spatial distributions of erythromycin resistance gene (*erm*B and *erm*F) concentrations in beef cattle backgrounding environment over a period of two years after cattle removal.

**Fig 4:**
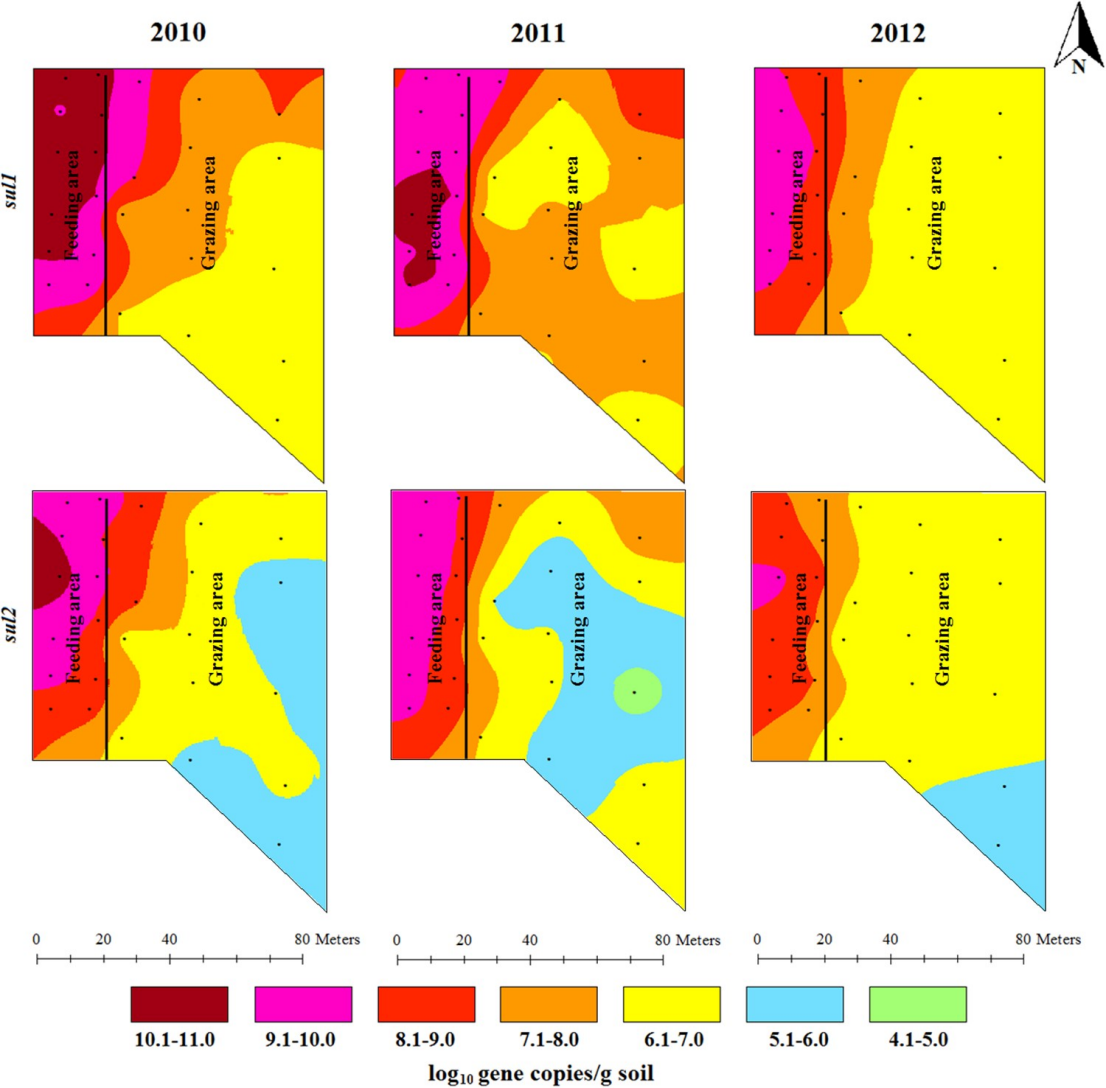
Spatial distributions of two sulfonamide resistance genes (*sul*1 and *sul*2) concentrations in beef cattle backgrounding environment over two years after cattle removal.

**Fig 5:**
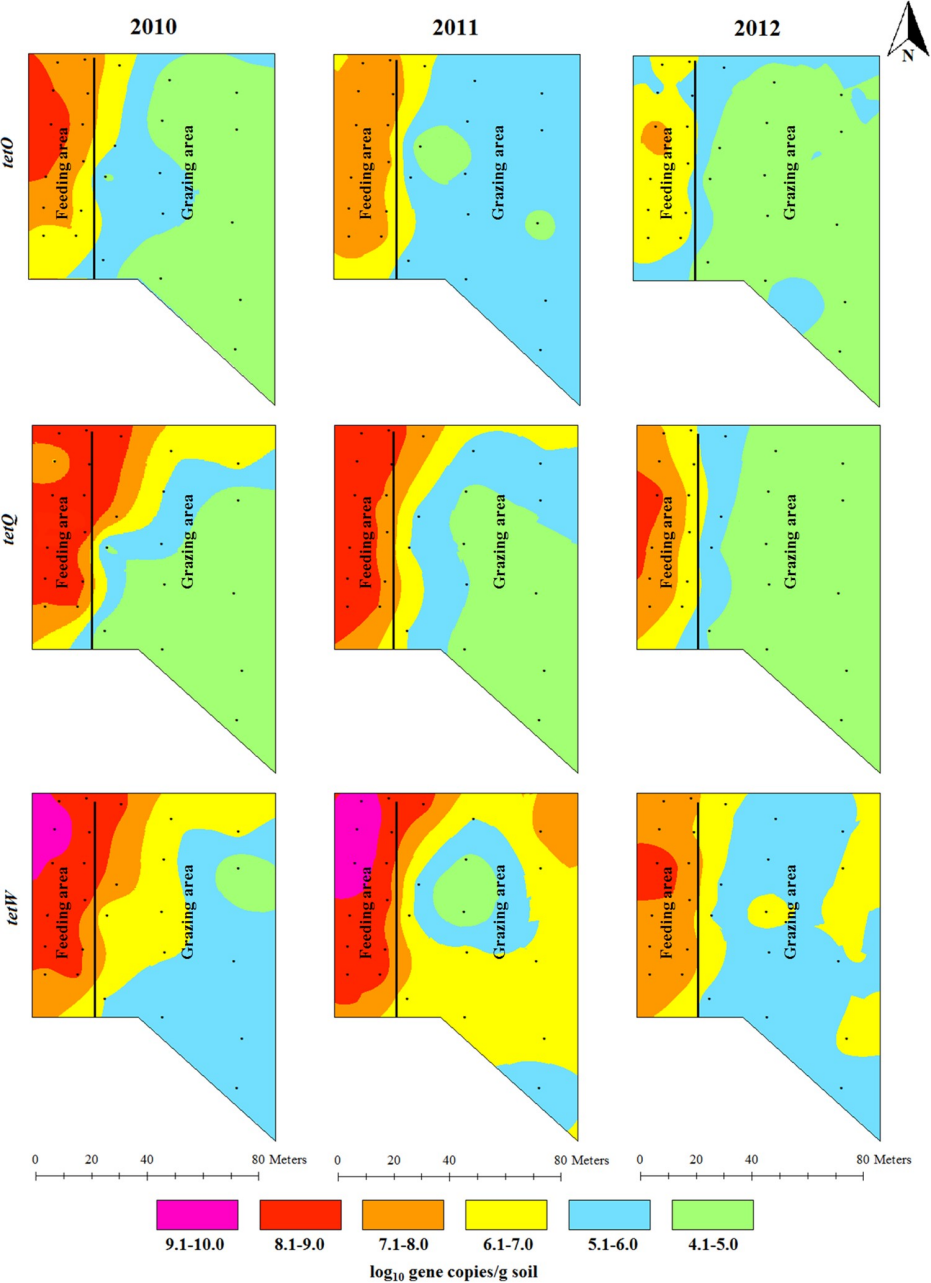
Spatial distributions of three tetracycline resistance genes (*tet*O, *tet*W and *tet*Q) concentrations in beef cattle backgrounding environment over a two years period after cattle removal.

The concentrations of *erm*B and *erm*F showed a gradient decline from their highest concentrations in the feeding area along the backgrounding landscape with diverse concentration ranges in 2010 and 2011. Higher concentration ranges (6.1–7.0 log_10_ gene copies/g) of *erm*B in 2012 were confined to the feeding area and around the fence in the grazing area. The concentration of *erm*F in 2012 still covered five concentration ranges (generally ranging from 4.1 to 9.0 log_10_ gene copies/g) showing its spatial concentration variability (Fig 3). The concentration of sulfonamide resistance genes was also spatially distributed across the backgrounding landscape with the highest concentration around the feeders and waterers in the feeding area, and the lowest concentration in the grazing locations farthest from the feeding area (Fig 4). The concentrations of tetracycline resistance genes showed a spatial gradient (Fig 5). While the spatial distribution in the concentrations of *tet*O and *tet*Q diminished over time, the spatial variation for *tet*W remained unchanged, despite decreases in *tet*W gene copies across all areas.

The concentrations of the seven ARGs studied were higher in the feeding area compared to the grazing area (S2 Fig) regardless of the sampling time. In the feeding area, one year after cattle removal the concentrations of the ARGs did not significantly (*P* > 0.05) change compared to the 2010 baseline level (Table 2; S2 Fig). However, two years after cattle were removed, the concentrations of ARGs in the feeding area significantly (*P* < 0.05) reduced ranging in magnitude from 1- to 1.5-log_10_ gene copies/g of soil compared to the 2010 level (Table 2).

### Effect of soil nutrients on the level of total bacteria and ARGs

Effects of soil nutrients, adjusted for the effects of yearly sampling time points and for the feeding and grazing areas of the backgrounding facility, on the concentrations of total bacteria, *Enterococcus* spp., class 1 integrons and the ARGs are shown in Table 3. Soil nutrient concentration was not significantly (*P* > 0.05) associated with total bacterial concentration. Only total carbon and ammonium concentrations were associated with increased concentrations of *Enterococcus* spp. The concentration of *int*I1 was not significantly (*P* > 0.05) associated with the concentration of any of the soil nutrients. For the ARGs, only ammonium concentration was significantly (*P* < 0.05) associated with increased concentrations of *erm*B and *tet*O.

**Table 3:** Effect of soil chemical measurements on the concentration of bacteria and antibiotic resistance genes in beef cattle backgrounding operation. Results are adjusted for sampling periods and areas (feeding and grazing areas) within the backgrounding operation.

	Coefficient	Standard error	t	P>|t|	95% Confidence interval	
**16s**						
Carbon	-0.001	0.006	-0.16	0.875	-0.012	0.010
Nitrogen	0.025	0.073	0.35	0.729	-0.120	0.170
Ammonium	0.001	0.001	0.76	0.448	-0.001	0.002
Nitrate	0.001	0.003	0.27	0.787	-0.005	0.007
***Enterococcus* species**						
Carbon	0.026	0.012	2.13	**0.037**	0.002	0.051
Nitrogen	-0.150	0.163	-0.92	0.361	-0.474	0.175
Ammonium	0.006	0.002	3.42	**0.001**	0.003	0.010
Nitrate	0.010	0.007	1.46	0.15	-0.004	0.023
***int*I1**						
Carbon	0.004	0.013	0.27	0.789	-0.023	0.030
Nitrogen	-0.014	0.174	-0.08	0.937	-0.361	0.334
Ammonium	0.001	0.002	0.24	0.812	-0.004	0.005
Nitrate	0.004	0.007	0.55	0.587	-0.011	0.018
***erm*B**						
Carbon	-0.003	0.014	-0.23	0.816	-0.031	0.025
Nitrogen	0.180	0.185	0.98	0.332	-0.188	0.549
Ammonium	0.007	0.002	3.19	**0.002**	0.003	0.011
Nitrate	0.006	0.008	0.82	0.414	-0.009	0.022
***erm*F**						
Carbon	-0.004	0.015	-0.28	0.78	-0.034	0.026
Nitrogen	0.111	0.198	0.56	0.576	-0.284	0.506
Ammonium	-0.0004	0.002	-0.16	0.877	-0.005	0.004
Nitrate	0.007	0.008	0.83	0.41	-0.010	0.023
***sul*1**						
Carbon	0.005	0.012	0.43	0.666	-0.018	0.028
Nitrogen	-0.035	0.152	-0.23	0.820	-0.338	0.268
Ammonium	0.001	0.002	0.29	0.772	-0.003	0.004
Nitrate	0.005	0.006	0.83	0.408	-0.007	0.018
***sul*2**						
Carbon	0.007	0.014	0.5	0.617	-0.021	0.035
Nitrogen	0.006	0.184	0.03	0.972	-0.360	0.373
Ammonium	0.002	0.002	1.06	0.293	-0.002	0.007
Nitrate	-0.003	0.008	-0.36	0.723	-0.018	0.013
***tet*O**						
Carbon	0.017	0.009	1.83	0.072	-0.002	0.036
Nitrogen	-0.184	0.125	-1.48	0.143	-0.433	0.064
Ammonium	0.004	0.001	2.96	**0.004**	0.001	0.007
Nitrate	0.010	0.005	1.87	0.067	-0.001	0.020
***tet*Q**						
Carbon	-0.007	0.017	-0.43	0.668	-0.041	0.026
Nitrogen	0.149	0.221	0.68	0.502	-0.292	0.591
Ammonium	-0.004	0.003	-1.49	0.141	-0.009	0.001
Nitrate	0.005	0.009	0.54	0.588	-0.013	0.024
***tet*W**						
Carbon	0.007	0.013	0.58	0.566	-0.018	0.033
Nitrogen	0.002	0.169	0.01	0.989	-0.335	0.339
Ammonium	0.001	0.002	0.49	0.624	-0.003	0.005
Nitrate	0.001	0.007	0.11	0.914	-0.013	0.015

## Discussion

In this study we investigated the persistence of ARGs in the environment that had been used for seven years for beef cattle backgrounding. We monitored the backgrounding facility for two years, after cattle were removed from the facility, for the levels of ARGs of three antibiotic classes (tetracyclines, sulfonamides and macrolides) that are commonly used in beef cattle production. The pasture-feedlot type setup of the backgrounding facility, in which animals are typically gathered around the feed bunks and water troughs for feeding and watering, gave us a unique opportunity to compare the levels and persistence of ARGs between the two compartments in the same facility and under the same management practices. We found that the levels of ARGs remained consistently higher in the feeding and watering area than in the grazing area. Statistically significant, albeit biologically small, reductions in the concentrations of the ARGs were observed two years after cattle were removed from the backgrounding facility.

The impacts of food animal production on the occurrence of antimicrobial resistance in the environment can be due to excreted antibiotics, the release of antibiotic resistant bacteria and resistance genes, or nutrient enrichment through fecal deposition. Excreted antibiotics from treated animals (through feces and urine) can select for resistant bacteria in the environment [38]. In the treated animals antibiotics select for resistant bacteria which along with the resistance genes are released into the environment [39]. Once in the environment, antibiotic resistant bacteria can persist and multiply, and the ARGs can be horizontally transferred to resident environmental bacteria. In the present study even though we were not able to quantify the amount of antibiotics used in the cattle populations in the last seven years we noted that the calves which became sick received injectable antibiotics (ceftiofur, tilmicosin, enrofloxacin and florfenicol). Although it would be interesting to see the maintenance of genes encoding resistance to these antibiotics, we point out that the study was not designed a priori for this purpose. We also note that in a cow-calf herd similarly managed on pasture Agga et al [40] did not find significant association between antibiotics use and resistance even in the fecal samples directly collected from the animals. We further note that it is practically impossible to measure all ARGs in a given ecosystem by qPCR and we realize the selection bias introduced by investigators by this approach. To gain a more in-depth information shotgun metagenomic sequencing can be used although it has its own limitations as well. The cumulative effect of antibiotic use in the backgrounding facility over seven years of operation on the observed levels and persistence of ARGs is undeniable. However, it is unlikely that antibiotic use alone is a major contributing factor for the maintenance of ARGs in the soils at the backgrounding facility, even after cattle were removed, since antibiotics were typically given to individual animals as needed and less frequently as compared to mass medication practice at finishing feedlots. Quantification of antibiotic concentrations from the soil samples was beyond the scope of the present study. However, Netthisinghe et al. [17] quantified three other non-antibiotic veterinary drugs (monensin, lasalocid and doramectin) from soil samples collected from the same backgrounding facility and reported higher concentrations in the feeding and watering area when compared to other parts of the backgrounding facility.

Higher concentrations of bacteria and ARGs in the feeding and watering area compared to grazing area clearly indicate that nutrient supply in the form of concentrated fecal deposition in the feeding and watering area plays a significant role for the propagation of bacteria including the resistant population. In a similar study setup significantly higher concentration of total bacterial populations and specific bacterial species (*Enterococcus* spp., *E*. *coli*, *Bacteriodes* spp.) were found in the feeding and watering area as compared to the grazing area [17]. Animal manure deposited in the animal production facilities or when land applied provides necessary nutrients for bacterial growth thus enriching resident soil bacteria (both susceptible and antibiotic resistant bacteria and associated ARGs). Udikovik-Kolic et al. [41] demonstrated higher levels of antibiotic resistant bacteria and ARGs in soil amended with manure from dairy cows that were not treated with antibiotics when compared to soil amended with inorganic fertilizer. The authors concluded that manure amendment induced a bloom of resident antibiotic resistant bacteria in the soil by providing nutrients and other factors necessary for bacterial growth. A beef cattle study [16, 42] that investigated the effect of a onetime 5-day in-feed administration of chlortetracycline (CTC) on the occurrence of antibiotic resistance in cattle feces and pen surface environments, clearly showed the impact of nutrient enrichment through fecal deposition. The authors observed that after a temporary increase in tetracycline resistant *E*. *coli* population following CTC in-feed administration, there was no difference between treated and the control group. However, there was a significant increase in the tetracycline resistant *E*. *coli* population [16] and selected ARGs [42] over the course of the study (120 days) regardless of CTC administration both in the fecal samples collected from cattle, and pen surface samples. Surprisingly, the levels of tetracycline resistant *E*. *coli* population and targeted ARGs in the pen surface samples collected from pens left unoccupied by animals during the study were significantly lower than the pens surface samples collected from pens occupied with cattle (both with and without CTC treatment) and remained unchanged during the 120 days of the study period.

The grazing area (21,900 m^2^) covered a relatively larger area compared to the feeding and watering area (2,400 m^2^) as shown in Fig 1 and previously described [18]. The relatively larger area of the grazing area limits the nutrient availability since fecal deposition is spread over a larger area as opposed to the feeding and watering area where animals are concentrated for feeding and drinking [17]. Although limited nutrient availability in the form of fecal deposition slows bacterial propagation and further spread of resistant bacteria and ARGs [40], the present study showed that the levels of the ARGs in the feeding area did not decline to the level observed in the grazing area of the backgrounding facility two years after cattle were removed from the operation. We note that this observation is despite the implementation of two successive management strategies: manure removal after cattle removal followed by grass harvesting [18].

Concentration of animals such as during feeding and watering promotes bacterial transmission through direct animal-to-animal contact and through ingestion of feed and water contaminated by feces. This concentrates total bacterial population including the resistant bacterial fraction and the associated ARGs in the feeder and watering areas as shown by the gradual decline in the concentrations of bacteria and ARGs over the feedlot landscape (Figs 2–4). Gathering of animals and manure deposition at the feeding and watering area of the backgrounding facility may also promote horizontal transfer of ARGs in the bacterial community. This was evident particularly by the presence of integrase genes (*int*1) of class 1 integrons, the concentrations of which declined in a gradient fashion along the backgrounding facility landscape with the highest concentration being observed at the feeding and watering areas (Figs 2–4). While the total bacterial population remained unchanged after two years, total *Enterococcus* spp. showed significant reduction (Fig 2; S1 Fig). This is an ecological phenomenon whereby one bacterial species is replaced by other bacterial species, perhaps more native to soil, over time thus maintaining total bacterial population. In the context of metagenomic study, identifying specific bacteria carrying the ARGs is important with regards to propagation and spread of antibiotic resistant bacteria. However, functional metagenomics, and 16S and shotgun metagenomic sequencing for microbial profiling were beyond the scope of this project.

The concentrations of the chemical elements and compounds known to provide carbon and nitrogen for bacterial growth were not found to be significantly associated (adjusted for sampling year and feeding and grazing areas of the backgrounding facility) with the concentrations of the bacterial population or the ARGs measured with the exceptions of carbon and ammonium (Table 3). Carbon concentration was significantly associated with increased concentration of *Enterococcus* spp.; ammonium concentration was associated with increased concentration of *Enterococcus* spp., *erm*B, and *tet*O. The lack of apparent association between the soil elemental nutrients and bacterial concentration and their associated ARGs requires further study. However, it can be hypothesized that either these nutrients were not limiting in the feedlot soils or that pure elemental nutrients are not sufficient to support bacterial growth, and that more complex protein and carbohydrate sources such as manure deposition are required. Although the use of QACs also co-selects for antibiotic resistance, we did not investigate *qac* genes that confer resistance to QACs. The ammonium nitrogen gas and other soil characteristics were measured in the context of potential sources of carbon and nitrogen to support bacterial growth in the soil and not in the context of disinfection use as in quaternary ammonium compounds (QACs).

## Conclusions

Our study suggests that ARGs, once present, could persist in the soil environment for at least two years even in the absence of the animal production effect. The presence of *int*I1 indicates the potential for the horizontal transmission of ARGs in the environment. Identifying factors responsible for the maintenance and transmission of ARGs and bacteria in the environment in the absence of selective pressure from active antibiotic use is essential to mitigate the spread of antibiotic resistant bacteria. Higher concentrations of ARGs were observed in the feeding area compared to the grazing area of the backgrounding facility suggesting fecal deposition plays a significant role in the propagation, maintenance and spread of antibiotic resistant bacteria and associated resistance genes. Although removal of cattle from the backgrounding operation for two years resulted in a statistically significant reduction in the concentrations of ARGs, evaluating whether these reductions are also biologically significant requires risk assessment studies. However, it is not clear how many more years it would take for ARGs to decline to pre-animal production level. The concentrations of total bacteria, total *Enterococcus* species and class 1 integrons, and ARGs showed spatial variations within and between the feeding and grazing areas (Figs 2–4) of the backgrounding landscape. In general, their concentrations were higher in the feeding area and in its immediate vicinity (around the fence and the gate) which was then followed by a gradient decline along the feeding and grazing areas of the backgrounding environment. The highest concentrations were seen around the feeders and waterers in the feeding area. Additional research is needed to identify factors that contribute to the maintenance of antibiotic resistance and to devise mitigation strategies to eliminate risks associated with those populations.

## Supporting information

S1 Fig Temporal variations in the concentrations of total bacteria, enterococcus and integrase gene of class 1 integrons in beef cattle backgrounding environment over two years after destocking. (TIF)

S2 Fig Temporal variation of antibiotic resistance gene concentrations in beef cattle backgrounding environment two years after destocking. A) Erythromycin resistance genes (*erm*B and *erm*F); B) Sulfonamide resistance genes (*sul*1 and *sul2*); C) Tetracycline resistance genes (*tet*O, *tet*Q and *tet*W).
(TIF)
