# Supplementary figures and images for: Persistence of antibiotic resistance genes in beef cattle backgrounding environment over two years after cessation of operation
Source: PLoS One. 2019 Feb 15;14(2):e0212510. doi: 10.1371/journal.pone.0212510 (PMC6377141; doi:10.1371/journal.pone.0212510)

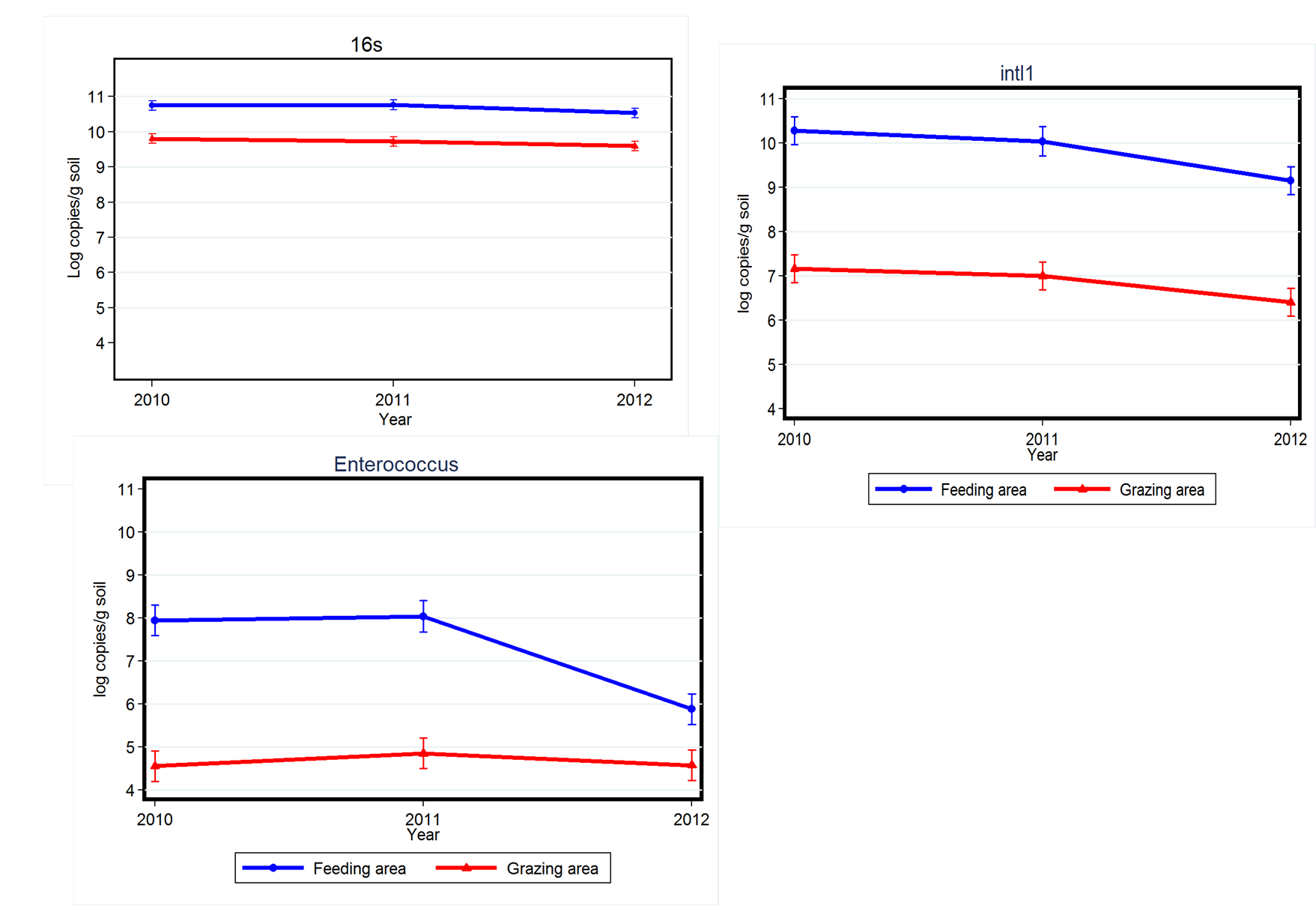

Supplement: S1 Fig — (TIF) [file pone.0212510.s001.tif]

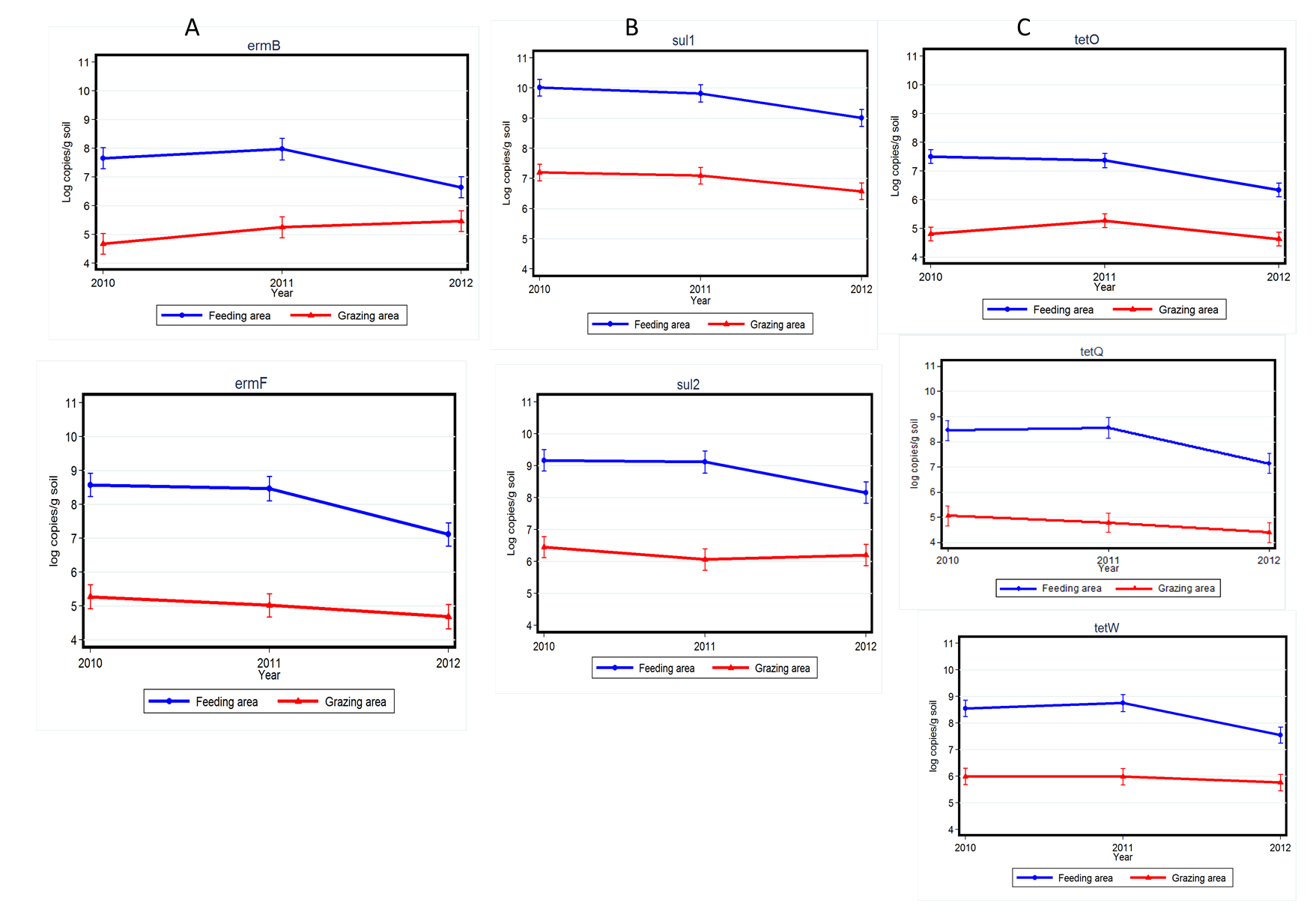

Supplement: S2 Fig — A) Erythromycin resistance genes (ermB and ermF); B) Sulfonamide resistance genes (sul1 and sul2); C) Tetracycline resistance genes (tetO, tetQ and tetW). (TIF) [file pone.0212510.s002.tif]
